# Impact of plasma 5-hydroxyindoleacetic acid, a serotonin metabolite, on clinical outcome in septic shock, and its effect on vascular permeability

**DOI:** 10.1038/s41598-021-93649-z

**Published:** 2021-07-08

**Authors:** Takeshi Tanaka, Masahiko Mori, Motohiro Sekino, Ushio Higashijima, Masahiro Takaki, Yoshiro Yamashita, Satoshi Kakiuchi, Masato Tashiro, Konosuke Morimoto, Osamu Tasaki, Koichi Izumikawa

**Affiliations:** 1 Infection Control and Education Center, Nagasaki University Hospital, 1-7-1 Sakamoto, Nagasaki, 852-8501 Japan; 2 Department of Infectious Diseases, Nagasaki University Hospital, 1-7-1 Sakamoto, Nagasaki, 852-8501 Japan; 3 Department of Paediatrics, University of Oxford, Oxford, OX1 3SY UK; 4 Division of Intensive Care, Nagasaki University Hospital, 1-7-1 Sakamoto, Nagasaki, 852-8501 Japan; 5 Department of Infectious Diseases, Nagasaki University Graduate School of Biomedical Sciences, 1-7-1 Sakamoto, Nagasaki, 852-8501 Japan; 6 Acute and Critical Care Center, Nagasaki University Hospital, 1-7-1 Sakamoto, Nagasaki, 852-8501 Japan

**Keywords:** Sepsis, Prognostic markers, Acute inflammation, Infection

## Abstract

Septic shock is characterized by dysregulated vascular permeability. We hypothesized that the vascular permeability of endothelial cells (ECs) would be regulated by serotonin via serotonin-Rho-associated kinase (ROCK) signaling. We aimed to determine the impact of 5-hydroxyindoleacetic acid (5-HIAA) on septic shock as a novel biomarker. Plasma 5-HIAA levels and disease severity indices were obtained from 47 patients with sepsis. The association between 5-HIAA levels and severity indices was analyzed. Permeability upon serotonin stimulation was determined using human pulmonary microvascular ECs. 5-HIAA were significantly higher in septic shock patients than in patients without shock or healthy controls (p = 0.004). These elevated levels were correlated with severity indexes (SOFA score [p < 0.001], APACHE II [p < 0.001], and PaO_2_:FiO_2_ [p = 0.02]), and longitudinally associated with worse clinical outcomes (mechanical ventilation duration [p = 0.009] and ICU duration [p = 0.01]). In the experiment, serotonin increased the permeability of ECs, which was inhibited by the ROCK inhibitor (p < 0.001). Serotonin increases vascular permeability of ECs via ROCK signaling. This suggests a novel mechanism by which serotonin disrupts endothelial barriers via ROCK signaling and causes the pathogenesis of septic shock with a vascular leak. Serotonin serves as a novel biomarker of vascular permeability.

## Introduction

Sepsis is a syndrome, defined as a life-threatening organ dysfunction caused by a dysregulated physiological host response to infection^1,2^. Sepsis complicated with shock status of hypotension was previously characterized as “severe sepsis” or “sepsis-induced hypoperfusion” in 1991 and 2001, respectively^2^. However, the existing variable definitions or terms (e.g., sepsis, severe sepsis, sepsis syndrome, septicemia, systemic inflammatory response syndrome) to recognize this syndrome have caused confusion in clinical practice. The new definition and clinical criteria of sepsis and septic shock was announced in 2016, as described in the “Methods” section^3^. Septic shock is characterized by dysregulated vascular permeability of endothelial cells (ECs); however, its detailed mechanism remains poorly understood. The incidence rate of sepsis and septic shock is 101.8 and 19.3 per 100,000 person-years, and the in-hospital mortality rate is 31.8% and 55.5%, respectively^4^. Clinically, the treatment outcome of sepsis and septic shock remains poor, and most patients require admission to the intensive care unit (ICU). Moreover, sepsis and septic shock causes a substantial financial burden^5^. This condition has an unpredictable clinical course, therefore, early diagnosis and treatment of sepsis (early goal-directed therapy 1-h bundle) should be performed, and public awareness regarding sepsis must be improved to prevent poor clinical outcomes^3,5^.

Serotonin (5-hydroxytryptamine [5-HT]) is a well-known neurotransmitter in the central nervous system (CNS) and is responsible for several physiological and behavioral processes such as mood, sleep, and cognitive functions^6^. 5-HT is synthesized by two different isoforms of tryptophan hydroxylase (TPH), TPH-1 and TPH-2. TPH-1 is expressed in the periphery whereas TPH-2 in the CNS^7^. Over 95% of 5-HT production in the whole body is executed in peripheral tissues, particularly in enterochromaffin cells in the intestine. Since the discovery of a dual system of 5-HT synthesis in the brain and periphery, a variety of pleiotropic functions of 5-HT in non-nervous tissues have been described, including its role in vasoconstriction^8^, proliferation^9^, and inflammation^10^. One of the major mechanisms that regulate vascular permeability is cellular contraction (clinically often discussed as vasoconstriction)^11^, which can be induced via the serotonin-RhoA/Rho-associated kinase (ROCK) signaling pathway in cell types such as vascular smooth muscle cells^12^.

Moreover, 5-HT is a pivotal immunological regulator in several diseases, such as autoimmune disorders, inflammatory bowel disease^13^, acute lung injury^14^, chronic obstructive pulmonary disease^15^, and pulmonary hypertension^12^. Platelets take up serotonin from plasma via serotonin transporters, which are known to be enriched in serotonin storage. They release serotonin during platelet activation, and the secreted serotonin plays a role in platelet aggregation and vasoconstriction^16^. Cloutier et al. showed that platelet-derived serotonin promotes endothelial vasculature leakage in an arthritis mouse model^17^.

Recently, we reported that macrophage phagocytosis dysfunction was regulated by 5-HT through Rho kinase activation^18^. For decades, Rho GTPase and ROCK, which are intracellular actin cytoskeletal organization regulated by Rho GTPase, have been studied extensively in phagocyte research. Phagocytosis is regulated by the balance between Rho GTPases (RhoA and Rac1), such that RhoA suppresses phagocytosis and Rac1 enhances phagocytosis. RhoA-mediated impaired clearance of apoptotic cells by macrophages leads to a host proinflammatory response and a delayed tissue repair^19,20^. For other cell types, there are already several reports demonstrating its modifying effect of ROCK inhibitor for endothelial dysfunction in human disease or animal disease models. Kinoshita et al. applied the injection of cultured human corneal endothelial cells (CECs) treated with a ROCK inhibitor in a CEC disorder patients^21^. Siddiqui et al. showed that ROCK1 inhibition protected against LPS-induced lung endothelial permeability. They confirmed its pathway in in vitro and in vivo experiments using p-MYPT1 and pp-MLC western blotting^22^.

Because of the growing body of evidence linking 5-HT signaling with cell contraction and the RhoA/ROCK pathway, we examined whether the vascular permeability of ECs would be regulated by 5-HT through Rho kinase activation. We also examined whether the 5-HT kinetics in sepsis patients reflecting vascular leakage conditions would increase, and if elevated kinetics play a role as an indicator of potential clinical severity.

Our study aimed to determine the impact of 5-hydroxyindoleacetic acid (5-HIAA), a serotonin metabolite, as a novel biomarker of sepsis severity, and to investigate the effect of 5-HT on vascular permeability.

## Results

### Baseline characteristics of the patients and healthy controls

The baseline clinical characteristics are shown in Table 1. Among them, a strong significant difference was observed in the SOFA scores between sepsis patients without shock and those with shock (median: 6 [interquartile range (IQR): 3–8] vs. median: 11 [IQR: 9–13], p < 0.001), suggesting that SOFA score would be a predominant clinical marker for evaluation of septic shock level.

**Table1:** Characteristics of patients and healthy controls.

Characteristics	Sepsis				Control
	Total	Shock (−)	Shock (+)	p	
Number (%)	47 (100)	14 (30)	33 (70)		7
Age (years)	76 (68–83)	73 (65–78)	77 (68–84)	0.200	34 (33.5–40)
Female (%)	18 (38)	2 (14)	16 (48)	0.040	2 (29)
SOFA score	10 (7–13)	6 (3–8)	11 (9–13)	< 0.001	
APACHE-II score	14 (10–19)	11 (7–16)	16 (11–19)	0.080	
PaO_2_:FiO_2_ ratio	238 (124–330)	273 (136–338)	214 (115–327)	0.600	
CRRT (%)	23 (49)	4 (29)	19 (58)	0.100	

The p values shown are the differences between the septic shock (−) group and shock (+) group: Mann–Whitney U-test for age, SOFA score, APACHE II score, and PaO_2_:FiO_2_ ratio, and Fisher’s exact test for sex distribution and CRRT ratio. Data are expressed as median (interquartile range).

*SOFA* sequential sepsis-related organ failure assessment; *APACHE* Acute Physiology and Chronic Health Evaluation; *PaO*_*2*_ partial pressure of arterial oxygen; *FiO*_*2*_ fraction of inspired oxygen; *CRRT* continuous renal replacement therapy.

### Serotonin metabolite kinetics in sepsis patients

**Figure 1:**
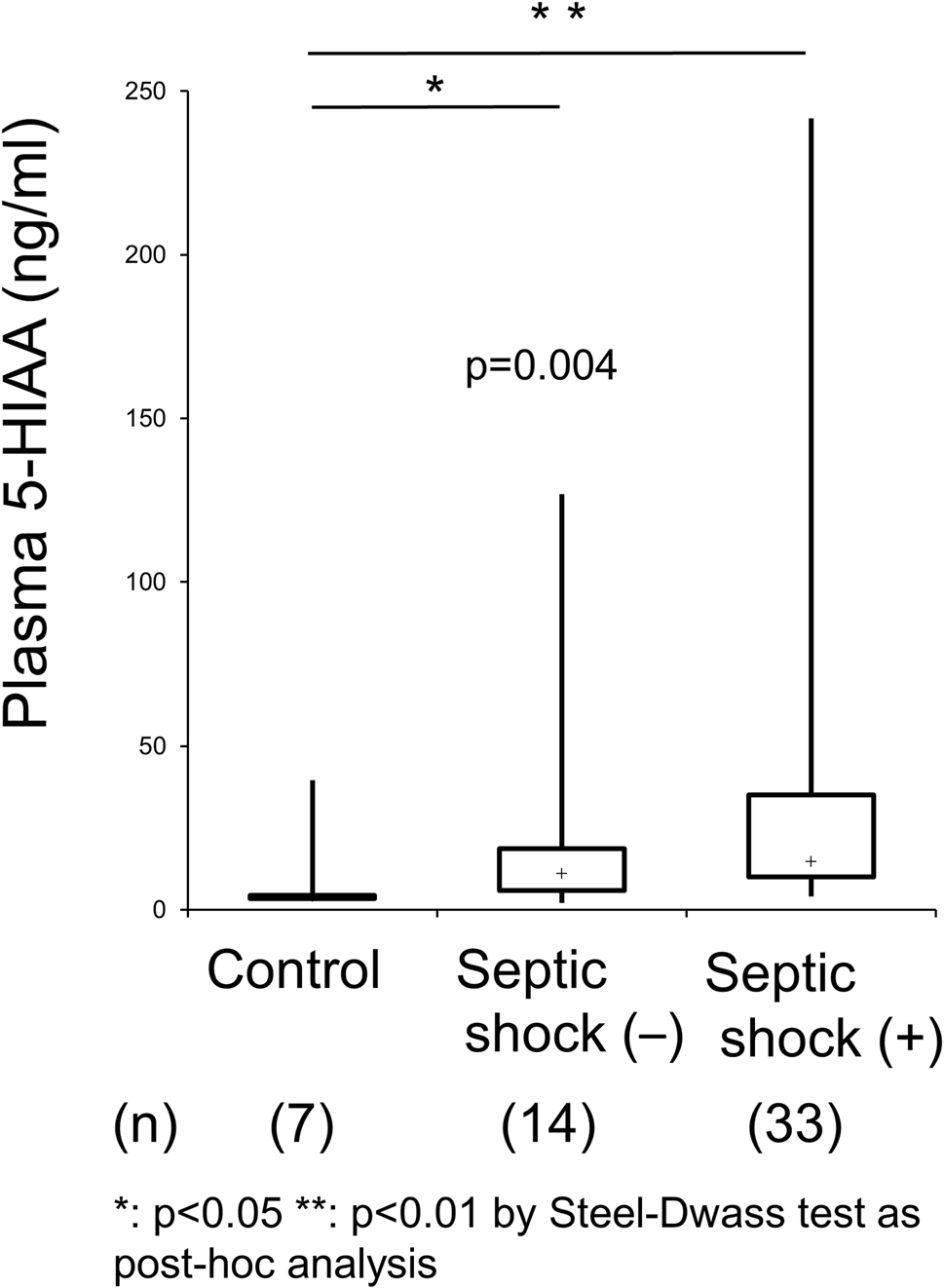
Plasma 5-HIAA levels in patients with septic infection. Plasma 5-HIAA levels among healthy controls, sepsis patients without septic shock, and sepsis patients with septic shock were determined by the Kruskal–Wallis test and Steel–Dwass test for post-hoc analysis. *p < 0.05, **p < 0.01 by Steel–Dwass test.

To investigate the associations between septic level and serotonin metabolite kinetics, we first analyzed the differences in plasma 5-HIAA levels between healthy controls and patients with sepsis. Plasma 5-HIAA levels were significantly higher in patients with sepsis than in healthy controls. In detail, these higher plasma 5-HIAA levels among septic patients were also identified in patients with septic shock (median: 14.7 ng/mL; IQR: 10.1–35.1 ng/mL), followed by sepsis patients without shock (median: 11.1 ng/mL; IQR: 5.8–19 ng/mL), and healthy controls (median: 4.1 ng/mL IQR: 3.4–4.5 ng/mL) (p = 0.004 by Kruskal–Wallis test, and p < 0.01 between septic shock and control, and p < 0.05 between sepsis without shock and control as post-hoc analysis) (Fig. 1). These results suggest a strong association between sepsis levels and plasma 5-HIAA levels.

### Correlation between disease severity indices and serotonin metabolite kinetics

**Figure 2:**
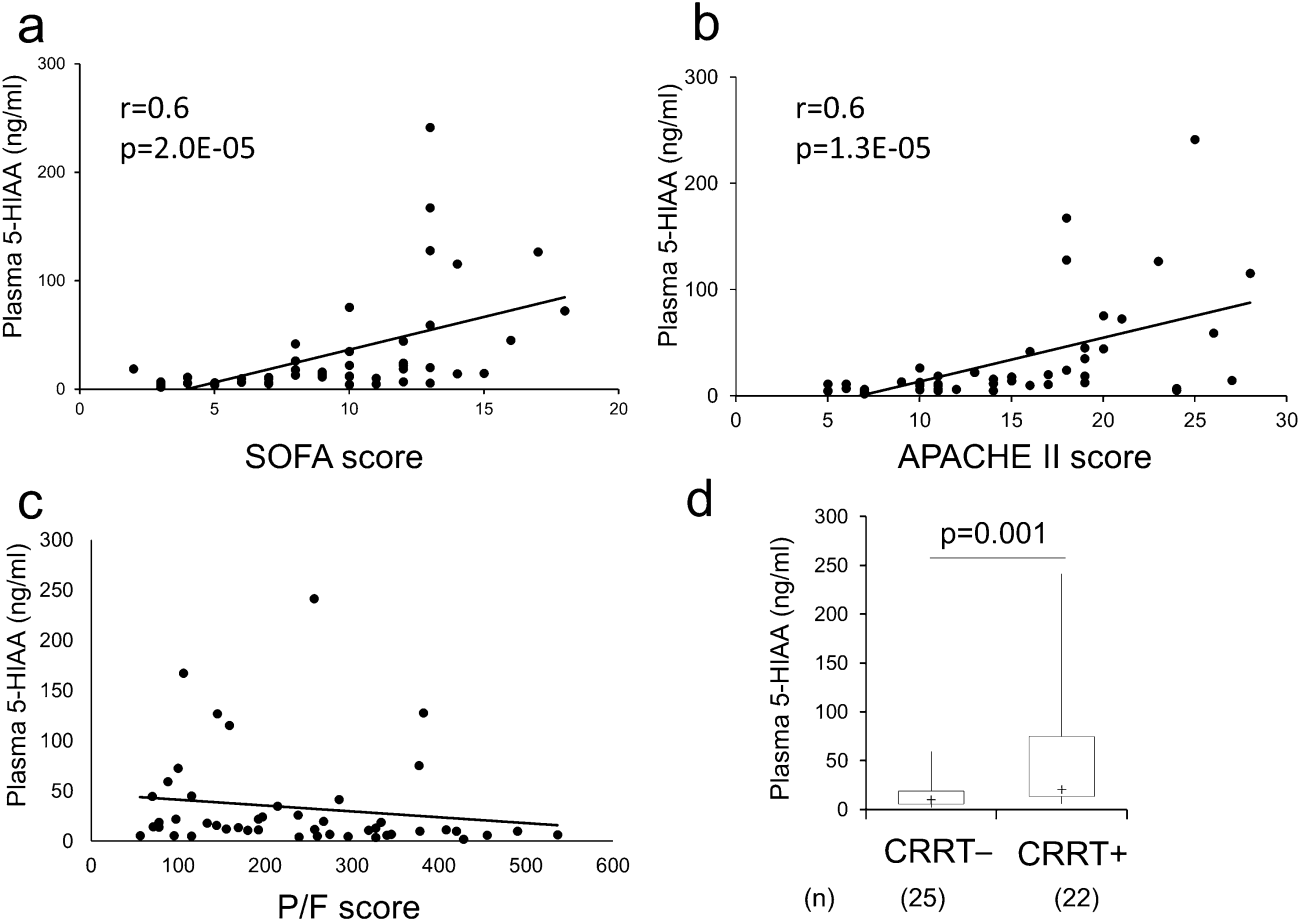
Correlation between disease severity indices and plasma 5-HIAA levels. Correlation between SOFA score and plasma 5-HIAA level (**a**); between APACHE-II score and plasma 5-HIAA level (**b**); and between PaO_2_:FiO_2_ ratio and plasma 5-HIAA level (**c**), by Spearman’s correlation test, and the plasma 5-HIAA level difference between sepsis patients without CRRT and sepsis patients with CRRT (**d**) by Mann–Whitney U-test.

Next, we analyzed the correlation between the disease severity indices and serotonin metabolite kinetics. Plasma 5-HIAA levels were positively correlated with clinical disease severity indices, such as SOFA score (r = 0.6, p < 0.001) (Fig. 2a) and APACHE II score (r = 0.6, p < 0.001) (Fig. 2b), and negatively correlated with PaO_2_:FiO_2_ ratio (r = 0.3, p = 0.02) (Fig. 2c). Sepsis patients with continuous renal replacement therapy (CRRT) had significantly higher plasma 5-HIAA levels than sepsis patients without CRRT (median: 20.7 ng/mL [IQR: 13.4–74.9 ng/mL] vs 10.1 ng/mL [IQR: 5.7–18.9 ng/dL]; p = 0.01) (Fig. 2d). These results revealed that plasma 5-HIAA levels could be a potential strong indicator to detect disease severity at the early stage of sepsis infection.

### Longitudinal analysis: worse clinical outcome among sepsis patients with higher plasma 5-HIAA level

**Figure 3:**
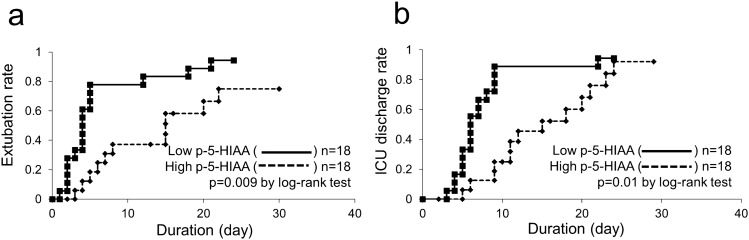
Longitudinal analysis of plasma 5-HIAA level and clinical outcome. The differences in the rate of mechanical ventilator extubation (**a**) and rate of ICU discharge (**b**) between the lower plasma 5-HIAA level group (≤ 13.6 ng/mL, n = 18) and higher-level group (> 13.6 ng/mL, n = 18). The results of the log-rank tests are shown.

Next, we assessed the impact of plasma 5-HIAA levels on longitudinal disease progression. A total of 36 out of 47 sepsis patients were admitted to the ICU and attached to a mechanical ventilator. After dividing the 36 patients into the lower plasma 5-HIAA level group (≤ 13.6 ng/mL, n = 18) and higher level group (> 13.6 ng/mL, n = 18), we assessed the differences in the clinical outcomes between the two groups. Compared with the lower plasma 5-HIAA level group, mechanical ventilator extubation (p = 0.009) (Fig. 3a) and ICU discharge (p = 0.01) (Fig. 3b) were delayed in the higher plasma 5-HIAA level group. These findings suggest that plasma 5-HIAA levels can be used to estimate the clinical progression of sepsis.

### Effect of serotonin on ECs permeability via ROCK pathway

**Figure 4:**
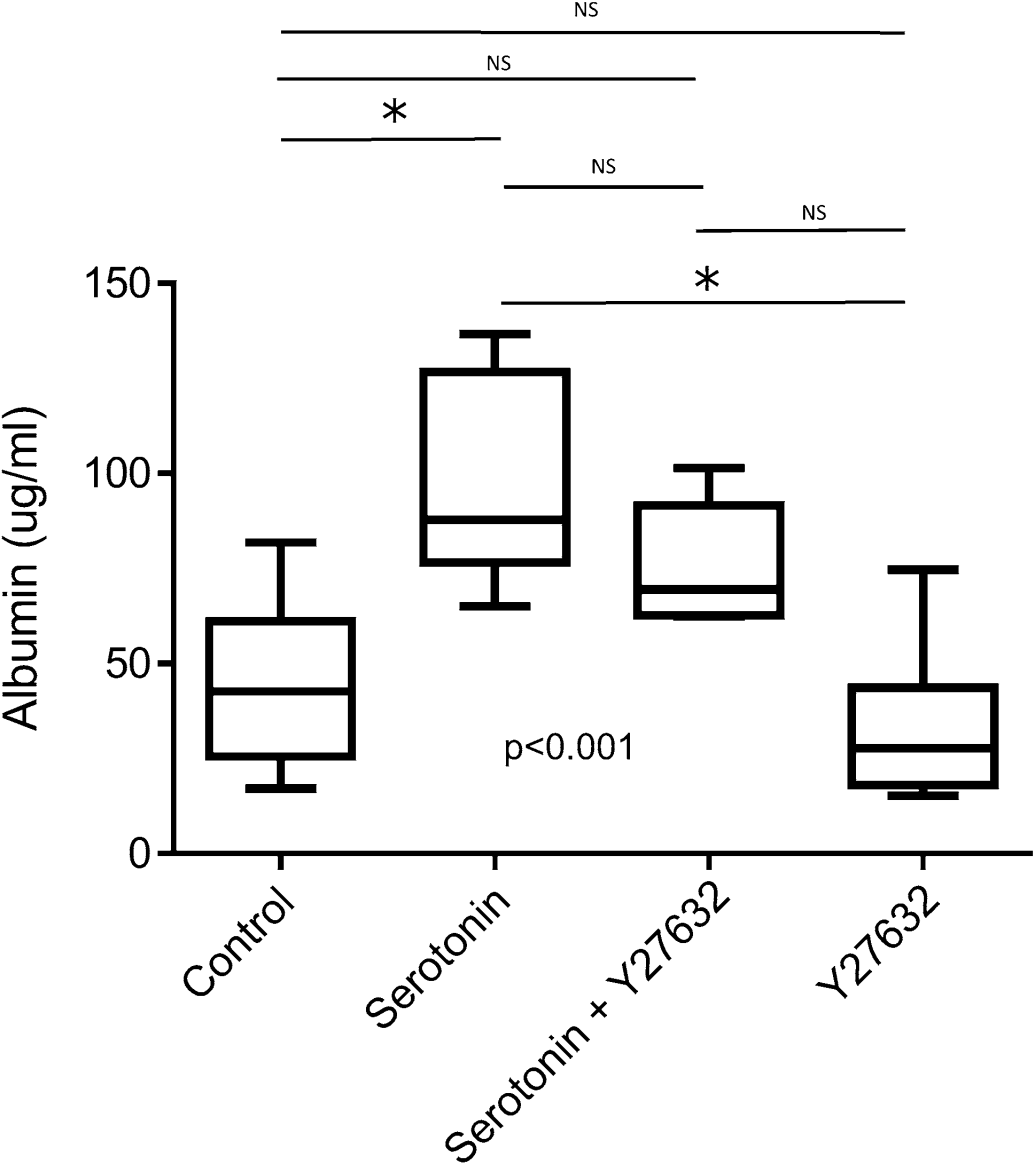
Effect of 5-HT on endothelial cell permeability. Albumin differences among control, serotonin-added, serotonin and ROCK inhibitor (Y27632)-added, and ROCK inhibitor-added groups by Kruskal–Wallis test and Steel–Dwass test for post-hoc analysis, are shown. *p < 0.05 by Steel–Dwass test.

Finally, to investigate the effect of 5-HT on EC permeability, we performed in vitro experiments to analyze the permeability of 5-HT-mediated ECs using HPMECs. A significant difference in albumin levels was observed among the four conditions (p < 0.001) (Fig. 4). Compared with control, serotonin stimulation significantly increased the albumin level (median: 42.7 μg/mL [IQR: 30.4–53.7 μg/mL] vs 87.8 μg/mL[ IQR: 77.4–108.6 μg/mL]: p < 0.01), with less increase in serotonin levels despite the activation of ROCK inhibitor (median: 42.7 μg/mL [IQR: 30.4–53.7 μg/mL] vs 69.4 μg/mL [IQR: 64.4–82.1 μg/mL]; p < 0.05). Our results suggest that the effect of 5-HT on EC permeability occurs through the ROCK activation pathway.

## Discussion

This study systematically investigated the effect of plasma 5-HIAA levels on the clinical outcomes of sepsis infection. In both cross-sectional and longitudinal analyses, we identified associations between higher plasma 5-HIAA levels and worse clinical outcomes of sepsis. We then addressed in the in vitro experiment the potential role of 5-HT in vascular permeability through the ROCK activation pathway, thereby suggesting a pivotal therapeutic target for vascular leak status in septic shock. Plasma 5-HIAA levels could be a novel biomarker of vascular permeability other than blood pressure and fluid resuscitation volume.

Due to the lack of evidence-based comprehensive treatment guidelines, septic shock treatment and its management are used as symptomatic treatments, which involve the use of fluid resuscitation, vasopressors, and antibiotics. However, septic shock remains a major health care burden in many countries^1–3^. Several studies have used novel treatments and management for patients with septic shock for (1) modulation of the hemodynamic response with the use of vasopressors (norepinephrine, epinephrine, phenylephrine, and vasopressin/terlipressin)^2^, and (2) modulation of the immunologic state with the use of CRRT^23,24^, *polymyxin* B hemoperfusion^25^, activated protein C^26,27^, and glucocorticoid therapy^28,29^. However, their impact on septic shock treatment is limited. Although supportive/symptomatic treatments are provided to septic shock patients, there is no specific or effective treatment or medication for this condition. These findings also imply the complex pathogenesis of sepsis and septic shock.

Regarding the pathogenesis of sepsis, Zhang et al. demonstrated the importance of 5-HT in driving sepsis mortality using an animal peritonitis model^30^.

The reasons we decided to measure plasma 5-HIAA instead of serotonin directly are as follows: (1) 5-HIAA is a metabolite of serotonin and is more stable than serotonin itself, therefore would be more favorable for septic shock level evaluation in hospitals. (2) HPLC or ELISA: Measurement of 5-HIAA by HPLC has already been established for clinical diagnosis such as carcinoid tumors. In addition, costly HPLC is more reasonable than ELISA; in other diseases, Hynes et al. reported an association between sudden infant death syndrome and elevated serum serotonin or 5-HIAA levels, as measured by ELISA or HPLC. These data suggest that 5-HIAA level measurement by HPLC would be available for disease severity evaluation of septic shock as well^31^. (3) Plasma or urine: Several septic shock patients enrolled in this study had complicated acute kidney failure and anuria, which made it difficult to collect stable urine samples for examination. Based on the above reasons, we assessed plasma 5-HIAA levels. Several benefits of plasma 5-HIAA measurement as a novel septic shock biomarker are considered.

In this study, we identified associations between higher plasma 5-HIAA levels and worse clinical outcomes of sepsis. Moreover, our in vitro assays, including ROCK inhibitors, also showed the effect of 5-HT on EC permeability through the ROCK activation pathway. These results were consistent with those of the abovementioned mouse model, and imply the importance of 5-HT pathways in septic shock pathogenesis and indicate that plasma 5-HIAA level is a novel biomarker for the evaluation of sepsis or septic shock severity.

The RhoA/ROCK pathway has been intensively studied considering its potential therapeutic effect by targeting ROCK inhibition. Clinical studies with systemic administration of ROCK inhibitor were conducted among patients with various diseases such as stroke^32^, pulmonary hypertension^33^, and angina^34^, which showed the beneficial effect of ROCK inhibitor on disease control. In infectious diseases, viral hemorrhagic fever diseases are commonly characterized by an increase in vascular permeability^35,36^. In terms of viral hemorrhagic fever disease, Eisa-Beygi et al. reported the potential feasibility of ROCK blockage as a therapeutic approach for Ebola disease^37^. In septic shock, a sepsis-related mouse model with ROCK inhibitor was used, which also showed a protective effect against sepsis^38,39^ or lung injury vascular permeability^22^. We applied this ROCK inhibition approach to our in vitro experiment, and our results also support the potential of ROCK inhibitor as a novel treatment for septic shock. Indeed, ROCK inhibitors and SERT inhibitors have already been clinically approved and utilized for the treatment of certain diseases: the former for the treatment of vasospasm and subarachnoid hemorrhage complications^40^, and the latter for the treatment of depression^41^. Clinical trials using these inhibitors to reveal the effect in regulation of sepsis-related vascular permeability are warranted.

This research, however, is subjected to some limitations. The first is the restricted number of patients and samples enrolled. A larger sample size would be needed to account for the high variability observed in the measurements of plasma 5-HIAA levels. Obtaining informed consent from patients and families with severe symptoms, including septic shock, was one of the most challenging parts of this study. The second limitation concerns the fact that the complete mechanism of RhoA/ROCK associated vascular permeability regulation could not be evaluated. The following previously reported studies support our hypothesis and the data presented. Our previous study demonstrated the 5-HT signaling pathway in macrophages using RhoA inhibitor (C3 transferase) and ROCK inhibitor (Y-27632) and assessed the downstream signaling of ROCK by observing phospho-MYPT1^18^. Guilluy et al. demonstrated the direct involvement of the 5-HT/RhoA/Rho kinase signaling pathway in pulmonary artery smooth muscle cells using RhoA-GTP pulldown assay or G-LISA assay and phospho-MYPT western blotting^12,42^. Wu et al. demonstrated that serotonin decreased transepithelial electrical resistance and reduced the expression of tight junction proteins. He also concluded that serotonin disrupted epithelial barrier function by modulating the levels of tight junction proteins^43^. Although the target cells are different, the conceptual idea arose from these previous studies.

In addition, Rap1 is a small GTPase that regulates cell–cell adhesion. In a recent study, the pivotal role of the adaptor protein ras-interacting protein 1 (Rasip1) as a Rap1-effector was revealed in endothelial barrier function^44^. It is a positive effector of endothelial integrity, which is contrary to the negative effect of RhoA/ROCK. To reveal the whole mechanism of our clinical observations, several types of Rho-GTPases need to be evaluated to explain the context of endothelial integrity. Regarding our results, further experiments to see more detailed involvement of 5HT-RhoA signaling by assessing activated RhoA, and visual assessment by immunofluorescence of the EC monolayer would be needed.

This study investigated the association between plasma 5-HIAA levels and disease outcome in sepsis infection, and we identified that plasma 5-HIAA levels can be a predominant biomarker of septic shock severity. We also found a novel role of 5-HT in vascular permeability through the ROCK activation pathway. These findings will contribute to the development of novel therapies and management strategies for sepsis and septic shock. Since there are no methods to detect ROCK activation directly as a visual marker in clinical samples (body fluids), we propose plasma 5-HIAA as a promising biomarker of ROCK activation in the human body as a prognostic marker of vascular permeability in clinical settings. This concept can be applied to other diseases (e.g., acute respiratory distress syndrome from any etiology, including COVID-19) in which vascular leakage is critical.

## Methods

### Enrollment of patients and healthy controls

This observational study was conducted at the Nagasaki University Hospital between August 2016 and March 2018. Adult patients (aged 18 to 90 years) admitted to the Nagasaki University Hospital ICU, emergency ward (EW), or infectious disease (ID) ward who were diagnosed with sepsis were recruited for the study. Sepsis, septic shock definitions, and clinical criteria from the third international consensus definitions for sepsis and septic shock were used in this study^3^. Sepsis was defined as suspected infection and a SOFA score of more than 2 points, while septic shock was defined as requiring vasopressor therapy to meet the mean arterial blood pressure (MAP) target of ≥ 65 mmHg and lactate target level of ≥ 2 mmol/L despite adequate fluid resuscitation. Meanwhile, pregnant patients and immunocompromised patients (those receiving chemotherapy, with human immunodeficiency virus infection, or treated with immunosuppressive agents) were excluded. Seven healthy control volunteers and 47 patients with sepsis were recruited for this study. The Institutional Review Board at Nagasaki University Hospital approved the study (approval number: 16072514). Written consent was obtained from each participant or legally authorized surrogate. All researchers involved in this research read the "Declaration of Helsinki (revised in October 2013)" and "Ethical Guidelines for Medical Research for Humans (2014 Ministry of Education, Culture, Sports, Science and Technology / Ministry of Health, Labor and Welfare Notification No. 3).” Implement compliance.

Plasma from patients with sepsis at the time of enrollment (day 0) and healthy volunteers were collected and stored at − 80 °C. To assess 5-HT kinetics, the alternative plasma 5-hydroxyindoleacetic acid (5-HIAA) breakdown product^45^ of 5-HT was monitored. Samples were sent to the SRL laboratory in Japan, and the plasma levels of 5-HIAA were measured by high-performance liquid chromatography. The baseline characteristics of patients and their follow-up data including SOFA score, Acute Physiology and Chronic Health Evaluation (APACHE) II score, PaO_2_:FiO_2_ ratio, duration of ICU hospitalization, and ventilator treatment days were obtained.

### In vitro experiment of permeability analysis

EC permeability upon 5-HT stimulation of the endothelial monolayer was determined by albumin transfer using human pulmonary microvascular endothelial cells (HPMECs). HPMECs were obtained from Lonza (Basel, Switzerland) and cultured in EC basal medium MV2^Ⓡ^ (PromoCell, Heidelberg, Germany) supplemented with 5% heat-inactivated fetal calf serum (FCS), growth factors, and supplements provided in EC Growth Medium MV2 SupplementMix^Ⓡ^ (PromoCell, Heidelberg, Germany), 100 μg/mL streptomycin, and 100 U/mL penicillin in humidified 10% CO_2_ at 37 °C and used for experiments between the sixth and seventh passages. The ECs suspended in the culture medium were seeded on a 6.5 mm Transwell^Ⓡ^ with a 0.4 μm pore polycarbonate membrane insert (Corning, NY, USA) at a density of 4 × 105 cells/filter insert. The inserts were placed into a 24-well culture plate, where each well was filled with 2 mL of culture medium and incubated at 37 °C in a humidified 5% CO_2_ atmosphere. After one week of culture, the cells reached confluence, and permeability measurements were performed. We measured albumin transfer across a cultured EC monolayer. The cells were pretreated with 10 μM Y-27632^Ⓡ^ (Wako, Tokyo, Japan) for 30 min and then incubated with 1 mM serotonin (Sigma-Aldrich, MO, USA) for 30 min. Then, the treatment solution was aspirated, and 500 μl of PBS containing 0.1% bovine albumin was added to the upper chamber and placed in new wells, in which each well was filled with 700 μl of PBS only. After 15 min, the inserts were removed. The solution in the lower chamber was collected and the albumin concentration was measured using a Bio-Rad Protein Assay Kit (Bio-Rad, Hercules, CA, USA).

### Statistical analyses

Statistical analysis was performed using SPSS 21.0 (IBM, Armonk, NY, USA). The Mann–Whitney U-test was used for the analysis of plasma 5-HIAA level differences between patients with and without CRRT. To analyze the differences in the plasma 5-HIAA levels among healthy controls, sepsis patients without septic shock, and sepsis patients with septic shock, and to determine the albumin levels among control, serotonin-added, serotonin and ROCK inhibitor (Y-27632)-added, ROCK inhibitor-added groups, the Kruskal–Wallis tests with Steel–Dwass tests for post-hoc analysis were used. Spearman’s correlation test was used to analyze the correlations between disease severity indices (SOFA score, APACHE II score, or PaO_2_:FiO_2_ ratio) and plasma 5-HIAA levels. In a longitudinal analysis, a log-rank test was performed to analyze the association between plasma 5-HIAA level (median value) and duration of ICU hospitalization or ventilator treatment.

## Data Availability

All data are fully available without restriction.
